# A System of Oral Surgery

**Published:** 1884-05

**Authors:** 


### BIBLIOGRAPHICAL.

The following notices were prepared for the April number of JOURNAL but were inadvertently omitted by the printer.

A SYSTEM OF ORAL SURGERY.—Being a Treatise on the Diseases and Surgery of the Mouth, Jaws, Face, Teeth and Associate Parts. By James E. Garretson, M. D., D. D. S., Illustrated with numerous illustrations. Fourth edition thoroughly revised with additions. Publishers: J. B. Lippincott & Co., Philadelphia, 1884, Price, cloth, $8.00 ; sheep $9.00.

The value of this noted work is increased by the addition of a considerable amount of interesting matter on subjects not heretofore alluded to, and much has been added to many of the former chapters. Seventy-five new illustrations appear to increase the value of the present edition, the first four hundred and seventy pages of which embrace the anatomy of the head and face, dental pathology and dental surgery, while the remaining portion treats of such parts of oral surgery as defects of the palate, diseases of the antrum and jaws, neuralgia, tumors of the jaws, inflammation, salivary fistulæ, dislocations and fractures of the maxillæ, anæsthesia, etc., etc.

Although some of the views expressed in this work concerning the development of the teeth, dental caries, etc., may be at variance with the opinions of other writers, yet there are many who will uphold those of the author of this treatise. Dr. Garretson has added so much to the present edition that the work has assumed quite large dimensions and necessitated the use of smaller type, in order to accommodate the 1037 pages in one volume, which is a useful and interesting contribution to the text books of the dental profession.

